# A successful case of anesthetic management of awake craniotomy using remimazolam and flumazenil in an elderly patient

**DOI:** 10.1186/s40981-023-00663-3

**Published:** 2023-10-28

**Authors:** Takehito Sato, Kimitoshi Nishiwaki

**Affiliations:** Department of Anesthesiology, Nagoya University Graduate School of Medicine, 65 Tsurumai-cho, Showa-ku, Nagoya city, Aichi, 466-8550 Japan

To the editor

Awake craniotomy is less applicable to elderly patients because of delayed emergence, the risk of developing delirium, and sudden hemodynamic changes during awakening [1, 2]. Remimazolam has been used for the anesthetic management of awake craniotomy in recent years [3]; however, cases of elderly patients receiving anesthesia with remimazolam have not been reported.

A 78-year-old woman (height 147.7 cm; weight 47.2 kg) was diagnosed with a left anterior temporal lobe tumor. Preoperative examination revealed only hypertension but no apparent of cognitive dysfunction, and an awake craniotomy with the asleep-awake-asleep method was scheduled. General anesthesia was induced with remimazolam 12 mg/kg/h, remifentanil 0.15 µg/kg/min, and rocuronium 20 mg, followed by insertion of the I-gel #3. Anesthesia was maintained with remimazolam 0.3–0.7 mg/kg/h for maintaining BIS between 40 and 60. After completion of dura incision, flumazenil 0.5 mg was administered following confirming recovery of spontaneous respiration, increase in BIS > 75, and response to naming. As her consciousness level improved immediately from Richmond Agitation-Sedation Scale 2 to 0, the I-gel was removed. During the awake phase, the patient was able to perform language tasks without any adverse events, such as nausea, seizures, or agitation. After 108 min of the awake phase, general anesthesia was reinduced with remimazolam 12 mg/kg/h and remifentanil 0.1 μg/kg/min, and the I-gel was inserted. The surgery was completed without any complications.

For patient safety management, it is important to maintain a good quality of arousal in the awake phase [4, 5]. Propofol and remifentanil are short-acting anesthetic drugs generally used for high-quality wakefulness without agitation. Most patients undergoing awake craniotomy are < 65 years of age. This is because propofol does not have an antagonist, and propofol anesthesia may cause poor awakening in elderly patients undergoing awake craniotomy [6]. Furthermore, they are unable to complete the required language tasks adequately in the awake phase.

The awake craniotomy guidelines of Japan suggest [2] that “if the required tasks can be handled correctly, awake surgery can be performed in persons older than 65 years of age.” Therefore, some institutions have attempted awake craniotomy for elderly patients aged ≥ 65 years. Former report has indicated that dexmedetomidine (DEX) can be used to safely manage anesthesia in elderly patients with awake craniotomy [6]; however, DEX does not have an antagonist similar to propofol.

Remimazolam is an ultra-short-acting benzodiazepine that is as effective as propofol in the induction and maintenance of general anesthesia [7, 8]. And BIS values during remimazolam anesthesia have been reported to be higher than those with propofol [9]. This is because the EEG during the sedation period showed an increase in beta waves (13–25 Hz) in addition to the occurrence of alpha waves during remimazolam administration, which is thought to have led to these high BIS values [9].

Benzodiazepine sedatives have also been reported to have fewer cardiovascular depressant effects than propofol [8]. Thus, remimazolam appears to be suitable for anesthesia in the elderly. It is unique as its anesthetic effect can be antagonized by flumazenil, and its use was reported to be safe for the asleep-awake-asleep method of awake craniotomy [3, 4]. In this case, it was possible to obtain clear arousal with remimazolam and flumazenil during awake craniotomy without any complications, suggesting that remimazolam has the potential to be suitable for awake craniotomy anesthesia in elderly patients.

In conclusion, we reported successful anesthetic management of awake craniotomy with remimazolam and flumazenil in an elderly patient.

## Data Availability

Not applicable.

## Abbreviations

BIS Index: Bispectal index
RASS: Richmond Agitation-Sedation Scale
