# Correction: A successful case of anesthetic management of awake craniotomy using remimazolam and flumazenil in an elderly patient

**DOI:** 10.1186/s40981-023-00671-3

**Published:** 2023-11-06

**Authors:** Takehito Sato, Kimitoshi Nishiwaki

**Affiliations:** Department of Anesthesiology, Nagoya University Graduate School of Medicine, 65 Tsurumai-Cho, Showa-Ku, Nagoya City, Aichi, 466-8550 Japan

**Correction: JA Clin Rep 9, 71 (2023)**

**https://doi.org/10.1186/s40981-023-00663-3**

Following publication of the original article [1], the author reported that there is an error in the maintext

The sentence

“After completion of, flumazenil 0.5 mg was administered following confirming recovery of spontaneous respiration, increase in BIS > 75, and response to naming.”

should read

“After completion of dura incision, flumazenil 0.5 mg was administered following confirming recovery of spontaneous respiration, increase in BIS > 75, and response to naming.”

The original article has been corrected.
